# Spatiotemporal distribution of leptospirosis in the Espirito Santo State, Brazil

**DOI:** 10.1590/S1678-9946202567058

**Published:** 2025-08-25

**Authors:** Nicolas Brune-Gonçalves, Gustavo Brune Gonçalves, Lucas Prata Vicente, Fernando Maffioletti Ferrari, Leone Salomão Vieiras Dalla Bernardina, Bernardo Santos Roza, Luis Augusto Pereira, Cecília Schettino de Araújo, Joamyr Victor Rossoni, Clairton Marcolongo-Pereira

**Affiliations:** 1 Centro Universitário do Espírito Santo, Programa de Pós-Graduação em Ciências da Saúde, Colatina, Espírito Santo, Brazil

**Keywords:** Leptospirosis, Spatial analysis, Public health, Kernel density estimation, Geographic Information System, Disease surveillance, Brazil

## Abstract

Leptospirosis, a neglected zoonotic disease of global relevance, particularly affects populations in socio-environmentally vulnerable regions. In tropical countries such as Brazil, the prevalence of leptospirosis increases significantly during floods, increasing human exposure to contaminated environments. This study aims to investigate the spatiotemporal distribution and prevalence of confirmed leptospirosis cases in Espirito Santo State, Brazil, from 2020 to 2024. This ecological study used secondary data from the Espirito Santo State Health Department and population estimates from the Brazilian Institute of Geography and Statistics. Prevalence rates were calculated by municipality. Kernel density estimation was used to assess spatial clustering. A total of 344 confirmed cases were reported during the study period, with the highest prevalence in the Southern and Metropolitan mesoregions. Most cases occurred in urban areas and predominantly affected economically active men aged 20-59 years. These findings highlight the influence of socio-environmental determinants on leptospirosis distribution and reinforce the importance of geospatial tools in finding high-risk areas and supporting targeted public health strategies.

## INTRODUCTION

The transmission of leptospirosis, an acute febrile infectious disease caused by bacteria of the genus *Leptospira*, primarily occurs via direct contact with water or soil contaminated with the urine of infected animals, particularly rodents^1^. It represents a major public health concern due to its severity and transmission pathways, occurring more frequently in socio-environmentally vulnerable areas. Limited access to safe drinking water, inadequate sanitation, and poor solid waste management exacerbate disease spread by creating favorable conditions for transmission^2^.

In tropical regions, such as Brazil, leptospirosis remains a significant public health issue, especially during and after floods, which increase human exposure to contaminated environments^3^. Brazil deems leptospirosis as a notifiable endemic disease throughout its territory^4^. Several factors contribute to its persistence in urban and semi-urban areas, including rodent populations, improper waste disposal, and the prevalence of informal settlements^5^.

Brazil reported 42,310 confirmed cases of leptospirosis from 2007 to 2017, with an annual average of 3,846 cases^6^. Currently, no effective vaccine is widely available for human leptospirosis, making prevention largely dependent on minimizing exposure to contaminated environments. The disease can cause severe complications, such as acute kidney injury, hepatic dysfunction, myocarditis, and pulmonary hemorrhage. Diagnosis is typically based on characteristic clinical manifestations, supported by epidemiological history, and confirmed by laboratory testing^3^.

Given the complex transmission dynamics of leptospirosis and the significant role of socio-environmental determinants, spatial and temporal analyses are crucial to understand its distribution patterns. These tools are instrumental in finding high-risk areas and guiding effective public health strategies. This study aims to investigate the spatiotemporal patterns and prevalence of confirmed leptospirosis cases in Espirito Santo State, Brazil, from 2020 to 2024, using geographic information system (GIS)-based tools and density estimation to find high-risk areas and support targeted public health interventions.

## MATERIALS AND METHODS

### Ethics

Secondary, aggregated, and de-identified data was used in this observational ecological study, dispensing with approval by an institutional ethics committee, according to the Brazilian Resolution CNS Nº 510/2016.

### Study area and design

This study was carried out in the Espirito Santo State, southeastern Brazil, which is composed of 78 municipalities in four mesoregions: Metropolitan, Southern, Central, and Northern. An ecological design was adopted to assess municipal trends and geographic patterns using confirmed cases of leptospirosis aggregated by year and location.

### Data sources

Data on confirmed leptospirosis cases were obtained from the official surveillance records of the Espirito Santo State Health Department (SESA), which was accessed on February 13, 2025. Annual population estimates for each municipality were retrieved from the Brazilian Institute of Geography and Statistics (IBGE). The cartographic base for geospatial analysis was provided in shapefile format by IBGE and referenced the Geocentric Reference System for the Americas (SIRGAS 2000).

### Prevalence calculation

Prevalence rates were calculated by municipality and mesoregion using the following formula:


$$P=\frac{\text{ number of diseases cases }}{\text{ residente population }}\times 100,000$$

The annual prevalence rates were then compared across the four mesoregions to find temporal and regional variations.

### Geospatial analysis tools

All spatial analyses were conducted on QGIS (version 3.28). Confirmed cases were georeferenced using the centroids of each municipality because of the absence of individual-level location data. Kernel density estimation (KDE) was applied to evaluate the spatial intensity of the case distribution and find clustering patterns (hotspots) from 2020 to 2024.

### Cluster classification criteria

High-risk areas were defined based on KDE raster outputs. Regions classified as “hotspots” corresponded to the upper quartile of density values (represented by the darkest color shades in the density gradient). This qualitative classification has been adopted in similar ecological studies using KDE to interpret relative spatial intensity^7^.

No formal spatial cluster test (e.g., SaTScan or Getis-Ord Gi*) was applied because the primary objective was descriptive mapping rather than statistical inference.

### Justification of statistical method

KDE, a non-parametric method, is widely used in spatial epidemiology to find areas of case concentrations. It provides a smoothed estimate of case density over space without assuming any underlying probability distribution. This technique is particularly appropriate for exploratory studies that aim to visualize geographic trends and inform public health planning.

The selection of KDE in this study follows the precedent of prior research on leptospirosis spatial dynamics in Brazil^4^ and avoids potential biases associated with low-resolution or underreported data.

### Visualization and outputs

All maps and prevalence charts were created on QGIS and Microsoft Excel 365. Kernel density maps were generated for the cumulative 2020-2024 period, and annual maps were produced to illustrate the changes over time. The final outputs included prevalence comparisons by mesoregion, cumulative density surfaces, and spatial overlays for risk identification.

## RESULTS

Espirito Santo reported 344 confirmed cases of leptospirosis from 2020 to 2024 (an average of 68.8 cases/year). Most cases (64.16%) occurred in urban areas, followed by rural (30.64%), and peri-urban zones (0.29%). In total, 4.91% of cases had missing data on the infection zone. Regarding sex distribution, men accounted for most cases (79.77%), whereas women, for 20.23%. Individuals aged 20–39 and 40–59 years configured the most affected groups, each comprising 35.17% of the total cases.

**Figure 1:**
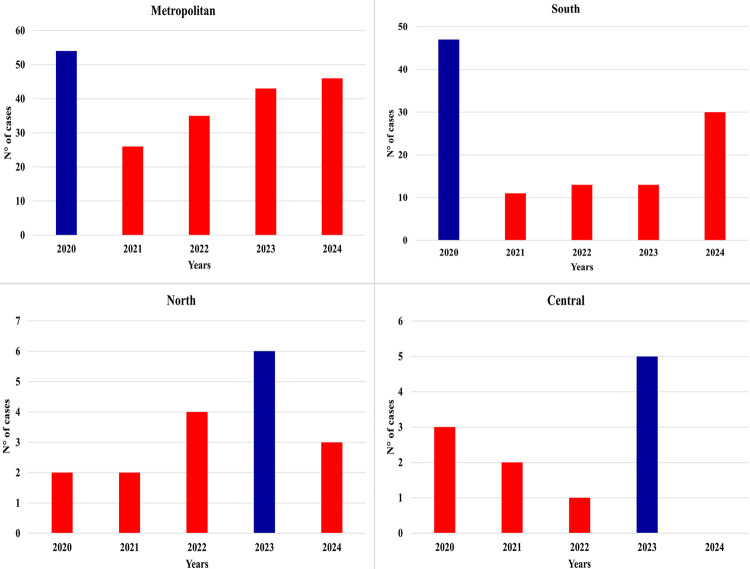
Annual distribution of confirmed leptospirosis cases by mesoregion in Espirito Santo State, Brazil (2020-2024).

Figure 1 shows the annual distribution of confirmed leptospirosis cases by mesoregion. The study period reported nine deaths associated with leptospirosis. In 2020, the Metropolitan and Southern mesoregions recorded the highest number of confirmed cases: 54 and 47 cases, respectively. Throughout the five-year period, the average annual number of cases in these regions totaled 40.8 and 22.4, respectively. In contrast, the Northern and Central mesoregions reported their highest annual case counts in 2023, with six cases each. The average annual number of cases in the Northern and Central regions totaled 3.4 and 2.2, respectively.

**Figure 2:**
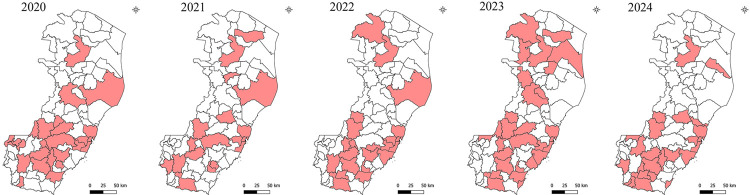
Spatial and temporal dynamics of leptospirosis in Espirito Santo State, Brazil (2020-2024).

**Figure 3:**
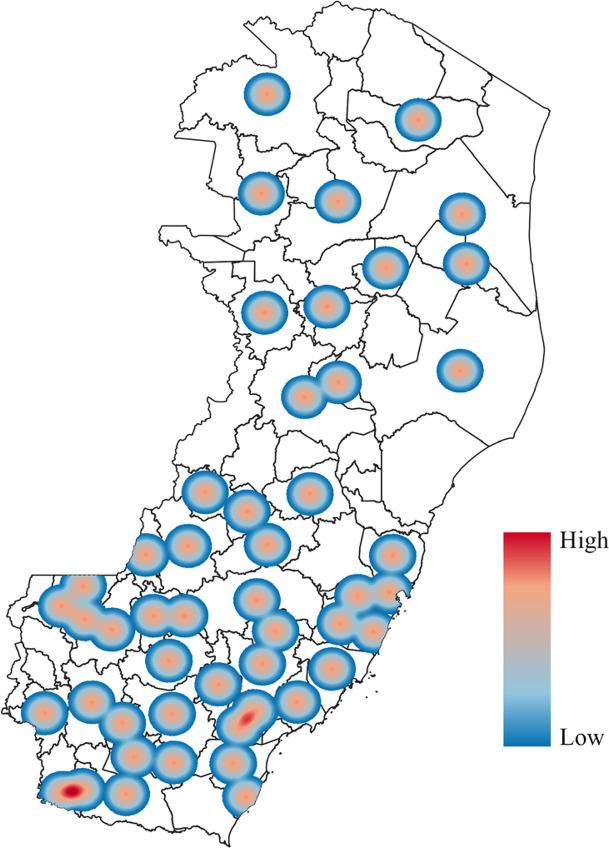
Hotspot analysis of leptospirosis prevalence in Espirito Santo State, Brazil (2020-2024).

**Figure 4:**
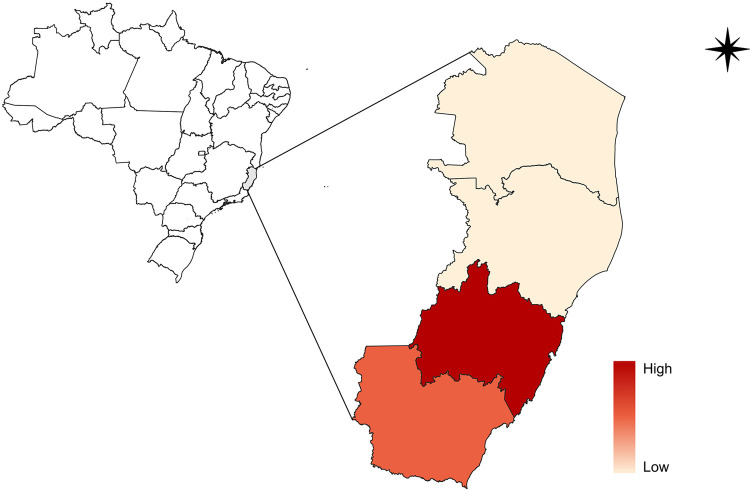
Estimated spatial concentration of leptospirosis cases in the mesoregions of Espirito Santo State, Brazil (2020-2024).

Figure 2 shows the spatiotemporal distribution of municipalities that reported at least one confirmed case during the study period. Of the 78 municipalities in the state, 50 registered at least one case each. Figure 3 shows the kernel density estimation of leptospirosis cases in the municipalities of Espirito Santo. Darker hues illustrate areas with a higher prevalence, with black representing the highest concentration (hotspots). The color gradient around these hotspots indicates a progressive decrease in the case density. Analysis showed persistent hotspots in the Metropolitan and Southern mesoregions across all years, suggesting that these regions are at greater risk of leptospirosis transmission. Figure 4 shows the spatial concentration of cases in the Metropolitan and Southern regions.

**Figure 5:**
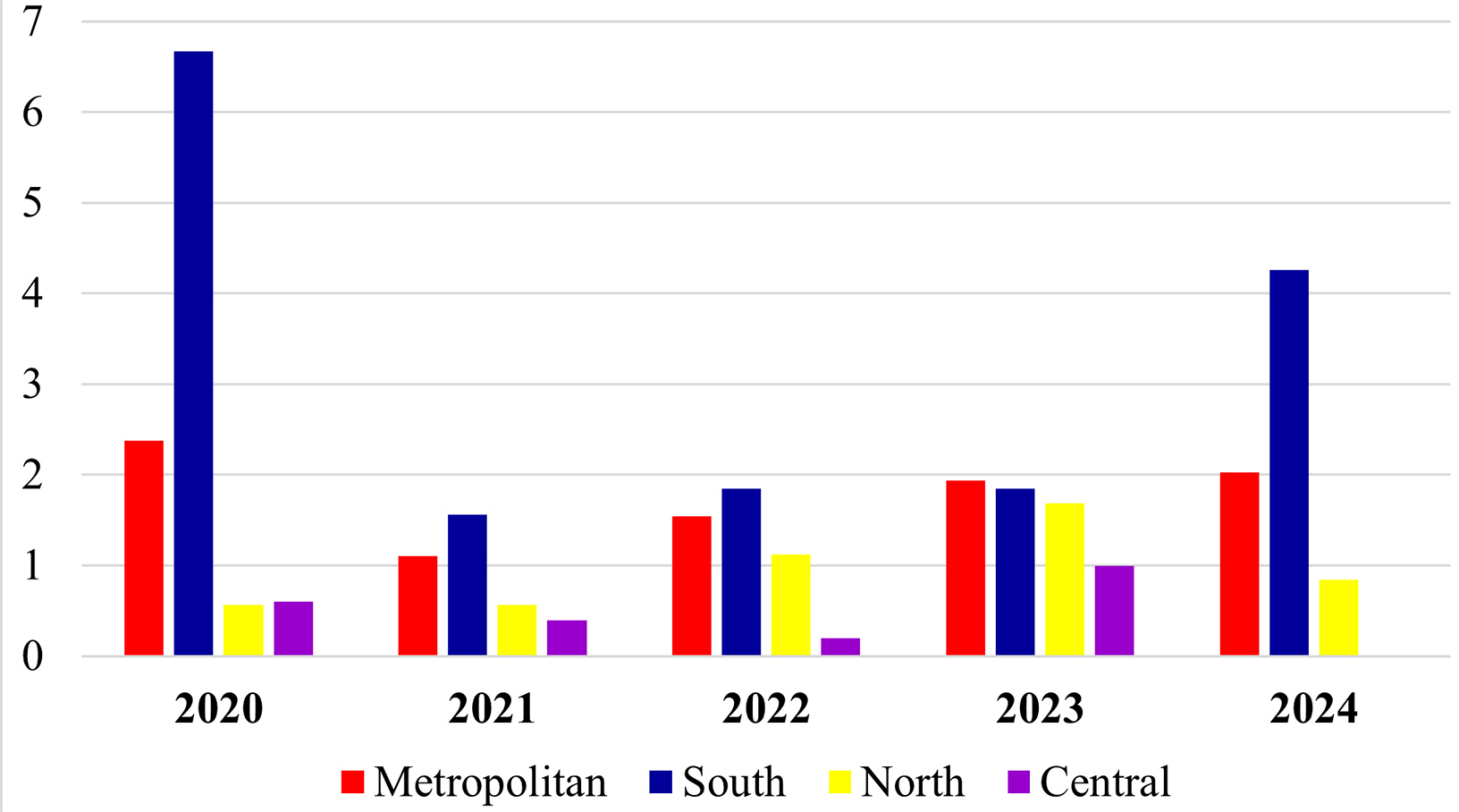
Annual prevalence of confirmed leptospirosis cases in Espirito Santo State, Brazil (2020-2024).

Figure 5 shows the annual prevalence rates by mesoregion, in which the Southern and Metropolitan regions showed the highest rates and notable peaks in 2020 and 2023.

## DISCUSSION

This study provides important insights into the spatial and temporal dynamics of leptospirosis in Espirito Santo State, Brazil, from 2020 to 2024. Of the 344 confirmed cases, the Metropolitan and Southern mesoregions showed the highest concentrations and prevalence, particularly in 2020. Although the Central and Northern mesoregions showed an increase in cases in 2023, their overall burdens remained low.

The observed regional disparities in leptospirosis distribution likely stem from a combination of socio-environmental and infrastructural factors. The Metropolitan region, for instance, shows high population density, disorganized urban expansion, and ongoing sanitation, drainage, and waste management issues; conditions that create an environment conducive to rodent proliferation and increase human exposure to contaminated water, especially during floods^8^.

Kernel density analysis confirmed persistent hotspots in urban centers, reinforcing the role of geography and infrastructure as key determinants of diseases. Although less urbanized, the Southern region also showed a high case burden. This may reflect the overlap between expanding urban areas with poor infrastructure and rural municipalities in which agricultural and livestock activities expose individuals to contaminated environments. These occupational exposures produce known risk factors in endemic regions^8^.

The predominance of urban cases (64.16%) agrees with prior research highlighting the role of environmental degradation, rodent infestation, and poor drainage in urban leptospirosis outbreaks^9,10^. On the other hand, the 30.64% of cases in rural areas suggest an additional transmission pathway linked to occupational exposure, particularly among agricultural workers^8,11^.

The COVID-19 pandemic may have caused the notable reduction in cases from 2021 to 2022. Restricted mobility, economic slowdowns, and temporary changes in environmental exposure patterns have likely impacted transmission. Moreover, health surveillance efforts targeted COVID-19, potentially contributing to the underreporting of leptospirosis^10^.

The demographic predominance of adult males (20-59 years) agrees with previous studies and supports the hypothesis of occupational exposure in high-risk environments as a central transmission driver^12^.

This study has several limitations. Reliance on secondary data from surveillance systems introduces the risk of underreporting and missing variables, such as occupation or comorbidities. The absence of georeferenced case data necessitated the use of municipal centroids for spatial estimation, which may reduce spatial precision. Additionally, ecological studies inherently limit causal inference at the individual level and are susceptible to fallacies.

Another constraint is the modest number of confirmed cases, which reflects the decision to use state-level surveillance data from SESA rather than from national systems such as the Notifiable Diseases Information System. Although the latter may report higher case counts, SESA data are locally validated and are considered more accurate and complete for regional analyses.

Despite these limitations, this study significantly contributes to a relatively understudied area. Few studies have addressed the spatiotemporal behavior of leptospirosis in Espirito Santo using geospatial tools. The use of GIS and kernel density estimation effectively found vulnerable areas and evaluated the geographic logic behind disease persistence.

These findings reinforce the utility of spatial analysis in public health surveillance. By showing territorial vulnerabilities, these tools can help guide resource allocation and support the design of geographically sensitive strategies to control leptospirosis. Integrating such methods into routine surveillance may strengthen disease prevention and response in endemic regions.

## CONCLUSION

This study showed the key spatial and temporal patterns of leptospirosis in Espirito Santo from 2020 to 2024. The disease was most prevalent in urban municipalities of the Metropolitan and Southern mesoregions, in which infrastructural deficits and socio-environmental vulnerability seemed to facilitate transmission.

The application of geospatial tools (including GIS and kernel density estimation) effectively found persistent hotspots and highlighted their territorial nature. These findings underscore the importance of integrating spatial analysis into epidemiological monitoring to improve public health strategies.

By focusing on localized vulnerabilities and the geographic logic of disease distribution, this study supports the development of intersectoral and regionally tailored interventions. Enhancing surveillance using spatial components may offer a more strategic and equitable approach to control leptospirosis in endemic contexts.
